# Geopolitics, climate change and health: what can we expect from the G20 Summit (Rio de Janeiro, Brazil, 2024)?

**DOI:** 10.1590/0102-311XEN068524

**Published:** 2025-01-13

**Authors:** Leandro Dias de Oliveira, Pablo Ibañez

**Affiliations:** 1 Universidade Federal Rural do Rio de Janeiro, Seropédica, Brasil.

On November 18th and 19th, 2024, Rio de Janeiro hosted the 19th meeting of the G20 Summit, attended by heads of State and Government of member countries and guests. Made up of 19 nations − Argentina, Australia, Brazil, Canada, China, France, Germany, India, Indonesia, Italy, Japan, Mexico, Russia, Saudi Arabia, South Africa, South Korea, Turkey, the United Kingdom and the United States − and two supranational bodies − the African Union and the European Union −, the G20 members represent about 85% of the world’s gross domestic product (GDP), more than 75% of trade, and about two-thirds of the world’s population.

Rio de Janeiro was chosen as the venue due to Luís Inácio Lula da Silva taking over presidency of the G20 on December 1, 2023, in a term that will last until November 30, 2024. Lula hosted the heads of State and Government, diplomatic delegations and representatives of international organizations, such as the International Monetary Fund (IMF) and the World Bank. There is an opportunity to promote Brazil as a positive leader globally: with a technical team strongly involved in solving problems and active participation of civil society, advances are expected in urgent agendas, such as addressing the impacts of climate change.

The meeting will take place one year before the 30th Conference of the Parties to the United Nations Framework Convention on Climate Change (COP 30), in Belém (Pará State, Brazil). Installed in the Amazon − a frontier perceived as a space to be preserved for the planets’ survival ^1^ −, delegations will be tasked with negotiating not only solutions to climate change, but also the implementation of multilateral actions that enable a less environmentally aggressive development model. There is a positive expectation for the COP 30 2025: after recent years of destroying environmental legislation and agencies in Brazil, the country will likely play a leading role in debates about protecting nature and opting for a more environmentally and socially righteous development.

Building a model that incorporates health, climate and economy, especially in the Amazon, involves a more integrated and cooperative approach between human, animal, plant, and environmental health ^2^; understanding relations between agrarian systems and their environmental and epidemiological impacts ^3^; strengthening technological development strategies for producing agroforestry systems and local productive arrangements ^4^, and creating indicators in different economic and social contexts ^5^.

A previous distrust about the effectiveness of meetings such as the G20 Summits and the climate related COPs exists, either due to mere disbelief in politics or to the inability of these major events to solve the discussed problems ^6^. Given how unlikely it is for structural changes to be made from the top down ^7^, rough edges and political difficulties for more robust advances have reverberated in fragile protocols and documents with elastic intentions on issues such as global warming.

Global warming is a geopolitical issue ^8^ that affects humans’ well-being. As early as 1990, the World Health Organization (WHO) detailed direct and indirect effects of climate change on health ^9^. Although significant gaps in academic literature that link health and climate change in South America ^10^ are still found, several studies and evidence proved this connection ^11^, reinforcing that the combination of poor sanitation, poverty and restrictions on access to health services can exacerbate effects of climate change on human health ^12^.

Increased morbidity and mortality from heat waves; increase of diseases due to poor access to clean water; increase in cardiovascular and respiratory diseases due to air pollutants; more groups facing food insecurity; increase in endemic infectious diseases such as malaria, leishmaniasis, leptospirosis and dengue are climate change impacts on health observed worldwide ^13^. Mental health disorders associated with natural disasters could also have climate change implications on human health ^14^.

Created in 1988, the Intergovernmental Panel on Climate Change (IPCC) was boosted with the signature of the United Nations Framework Convention on Climate Change (UNFCCC) ^15^ during Eco-92 and, five years later, with the *Kyoto Protocol* ^16^. At the same time, Brazil was stigmatized as a “world enemy of the environment”, which was observed in 1988 headlines of the New York Times, entitled *Vast Amazon Fires, Man-Made, Linked To Global Warming*, and in the editorial entitled *Who is Burning the Amazon?* ^17^, along with harsh North American and European summers, floods in Bangladesh and hurricanes in the Caribbean.

The North-South divide has always characterized the negotiating process of international environmental conferences ^18^: Northern countries are the greatest degraders of common goods and waste-producers, while Southern countries suffer the consequences and costs of this process. Forest distribution is also unequal, since peripheral nations are most susceptible to carbon markets under biodiversity preservation schemes.

This is a complex situation because it is necessary to reformulate the cornerstones of a global economy powered by fossil fuels − which directly impacts human health ^19^ −, but we live with the failure of mitigation policies and instruments. Thus, the urgency of adapting to climate change is increasing, as it is estimated that, by 2050, emissions would have to be 50% below 1990 levels and be zero or negative by 2100 ^20^.

The 2030 Agenda ^21^ has offered global policy coherence with explicit reference to health, economic development and climate change adaptation ^22^. Adaptations in cities can reduce urban heat islands and increase resilience to floods. Also, specific buildings standards can alleviate many climatic impacts, from energy consumption to the transmission of infectious diseases ^23^. In health, investment in research will provide ways to face the consequences of climate change, with measures that reconcile environment and public health ^24^. These health phenomena require preventive measures such as improving surveillance systems via satellite images and climate models able to generate alerts for emerging or reemerging infectious diseases ^25^.

In preparatory meetings for the G20, the participation of researchers from renowned institutions in developing the agendas has been promissed. Issue Notes, which are alignment documents between the G20 member countries with guidelines on the central themes, were also prepared. Climate, poverty, bioeconomy, agriculture, employment, education, health and energy transition are some of the listed topics.

In the Health Working Group’s Issue Note, there is a segment on climate change, reflecting on links between climate change and the emergence and spread of infectious diseases in urban peripheries, health impacts of heatwaves, access to clean water, and climate change impacts on mental health. Brazil, as India and Indonesia have previously done, reinforced the role of One Health, an integrative approach in which public health is no longer restricted to humans alone, but involves the whole environment ^26^.

The G20 Task Force on a Global Mobilization against Climate Change, define its priorities as follows: revise measures to mobilize private capital for developing countries; focus public and private funding on sustainable development and climate action; and encourage financial agencies to allocate resources for climate change mitigation and adaptation and to implement the 2030 Agenda ^27^.

A set of political-economic actions capable of combining energy transition, State action and socio-environmental justice on a global scale is needed. Climate change requires strong measures in public policies and dialogue with civil society. Trading of carbon credits, slightly lowering pollution indices or mobilizing investments without concrete counterparts and with flexible measurements are insufficient to build a promising scenario for coming years.

It remains to be seen whether the G20 Summit will be able to deliver concrete results. Inserted in a universe whose governance is based on multilateralism, debates on issues such as inequality, hunger and the environmental are proving to be fundamental. The G20 houses the G7, which brings together the most industrialized countries in the Western world and takes a tough stance on issues such as financing, economic growth, and wealth management. It is expected that, in Brazil, G20 measures will advance institutionally on pressing issues, such as climate. Creating protocols with a greater degree of concreteness and the agreed adoption of measures that expand global socio-environmental justice, with reliable and lasting financing articulations, will be a great encouragement for the COP 30, to be held in Belém in 2025.

The G20 must build a more effective institutionalism, including actions related to financial support in line with the urgency that the issue presents. To include health in the documents seems appropriate, but there is still a lack of clear mechanisms for action. Simple compensation as an environmental logic is a type of ecological indulgence for richer countries based on the purchase of the right to pollute ^28^. And even with corporate initiatives to clean up and anticipate environmental damage and with the action of environmental NGOs ^29^, if the total financialization of nature and rationalization of economic and environmental arrangements from a business point of view on a global scale prevail, improvements tend to be very timid.
