# Supplementary material for: Geopolitics, climate change and health: what can we expect from the G20 Summit (Rio de Janeiro, Brazil, 2024)?
Source: Cad Saude Publica. 2025 Jan 13;40(11):e00068524. doi: 10.1590/0102-311XEN068524 (PMC11729366; doi:10.1590/0102-311XEN068524)
Supplement: Translation

# Translation: Geopolítica, mudanças climáticas e saúde: o que podemos esperar da Cúpula do G20 (Rio de Janeiro, Brasil, 2024)?

**DOI:** 10.1590/0102-311XPT068524

**Authors:** Leandro Dias de Oliveira, Pablo Ibañez

**Affiliations:** 1 Universidade Federal Rural do Rio de Janeiro, Seropédica, Brasil.

Nos dias 18 e 19 de novembro de 2024, o Rio de Janeiro sediou a 19ª Reunião da Cúpula do Grupo dos 20 (G20), com a presença dos chefes de Estado e de Governo dos países-membros e convidados. Formado por 19 nações - África do Sul, Alemanha, Arábia Saudita, Argentina, Austrália, Brasil, Canadá, China, Coreia do Sul, Estados Unidos, França, Índia, Indonésia, Itália, Japão, México, Reino Unido, Rússia e Turquia - e dois órgãos regionais - União Africana e União Europeia -, os membros do G20 representam juntos cerca de 85% do produto interno bruto (PIB) mundial, mais de 75% do comércio e cerca de dois terços da população de todo o mundo.

A escolha da cidade do Rio de Janeiro como sede desse encontro se deveu ao fato de Luís Inácio Lula da Silva ter assumido a presidência do G20 em 1º de dezembro de 2023, num mandato que se estenderá até 30 de novembro de 2024. Lula foi anfitrião dos chefes de Estado e de Governo, das delegações diplomáticas e dos representantes de organismos internacionais, como o Fundo Monetário Internacional (FMI) e o Banco Mundial. Vislumbra-se a oportunidade de projeção do Brasil como liderança positiva no cenário global: com uma equipe técnica fortemente envolvida na solução de problemas e a participação ativa de atores da sociedade civil, são esperados avanços em pautas urgentes, como o enfrentamento dos impactos das mudanças climáticas.

A reunião ocorre um ano antes da 30ª Conferência entre as Partes da Convenção-Quadro das Nações Unidas sobre Mudança do Clima (COP-Clima), em Belém (Pará, Brasil). Instalados em plena Amazônia - uma fronteira percebida como espaço a ser preservado para a sobrevivência do planeta ^1^ -, as delegações presentes terão a missão de negociar não somente soluções para as mudanças do clima, mas também a implementação de ações multilaterais que permitam um modelo de desenvolvimento ambientalmente menos agressivo. Há uma expectativa positiva para a COP-Clima 2025: após os recentes anos de destruição das legislações e dos organismos ambientais no Brasil, espera-se o protagonismo do país nos debates sobre a proteção da natureza e a opção por um desenvolvimento mais justo ambientalmente e socialmente.

A construção de um modelo que integre saúde, clima e economia, especialmente na Amazônia, envolve uma abordagem mais integrada e cooperativa entre a saúde humana, animal, vegetal e ambiental ^2^, a compreensão das relações entre sistemas agrários e seus impactos ambientais e epidemiológicos ^3^, o fortalecimento de estratégias de desenvolvimento tecnológico para a produção de sistemas agroflorestais e arranjos produtivos locais ^4^ e a criação de indicadores em diferentes contextos econômicos e sociais ^5^.

Existe uma prévia desconfiança acerca da efetividade de encontros como as Cúpulas do G20 e as COPs-Clima, seja pela mera descrença na política ou pela incapacidade de eventos desse porte resolverem os problemas discutidos ^6^. Como é improvável que mudanças estruturais sejam realizadas de cima para baixo ^7^, as arestas e as dificuldades políticas para avanços mais robustos têm reverberado em protocolos frágeis e documentos com intenções elásticas em assuntos como o aquecimento global.

O aquecimento global é uma questão geopolítica ^8^ que afeta a humanidade. Já em 1990, a Organização Mundial da Saúde (OMS) detalhou os efeitos diretos e indiretos das mudanças climáticas na saúde humana ^9^. Ainda que haja lacunas significativas nas publicações acadêmicas que relacionem os temas na América do Sul ^10^, diversos estudos e evidências comprovaram essa relação ^11^, reforçando que a combinação de condições precárias de saneamento, a pobreza e as restrições de acesso a serviços de saúde podem agravar os efeitos das mudanças climáticas sobre a saúde humana ^12^.

São impactos da mudança climática na saúde em todo o mundo: aumento da morbidade e mortalidade por ondas de calor; ampliação na incidência de doenças devido à piora no acesso à água potável; aumento de casos de doenças cardiovasculares e respiratórias por causa de poluentes atmosféricos; elevação do contingente populacional em condições de insegurança alimentar; e aumento da incidência de doenças infecciosas endêmicas como malária, leishmaniose, leptospirose e dengue ^13^. Distúrbios de saúde mental associados a desastres naturais também poderão ser implicações ^14^.

O Painel Intergovernamental sobre Mudanças Climáticas (IPCC), criado em 1988, foi impulsionado com a assinatura da Convenção-Quadro das Nações Unidas sobre Mudanças Climáticas (UNFCCC) ^15^ durante a Eco-92 e com o *Protocolo de Kyoto* cinco anos depois ^16^. Ao mesmo tempo, o Brasil era estigmatizado como “inimigo mundial do Meio Ambiente”, algo observado nas manchetes do *The New York Times* em 1988, com o título *Grandes Incêndios na Amazônia, provocados pelo Homem, relacionados ao Aquecimento Global*, e no editorial intitulado *Quem está Queimando a Amazônia?* ^17^, junto aos rigorosos verões norte-americano e europeu, inundações em Bangladesh e furacões no Caribe.

A divisão Norte-Sul sempre caracterizou o processo negociador das conferências ambientais internacionais ^18^: os países do Norte são os maiores degradadores dos bens comuns e produtores de lixo, enquanto os países do Sul sofrem com as consequências e custos desse processo. A distribuição das florestas também é desigual, pois são as nações periféricas as mais propícias para o mercado de carbono sob esquemas de preservação da biodiversidade.

A situação é complexa porque existe a necessidade de reformulação dos fundamentos de uma economia global alimentada por combustíveis fósseis - que impactam diretamente a saúde humana ^19^ -, mas convivemos com o fracasso das políticas e dos instrumentos de mitigação. Dessa maneira, a urgência da adaptação às alterações climáticas aumenta, pois se estima que, até 2050, as emissões precisariam estar 50% abaixo dos níveis de 1990 e serem zero ou negativas em 2100 ^20^.

A Agenda 2030 ^21^ tem oferecido coerência política global com referência explícita à saúde, ao desenvolvimento econômico e ao ajuste à mudança climática ^22^. Adaptações nas cidades podem reduzir os efeitos das ilhas de calor e aumentar a resiliência às inundações, bem como os padrões de construção são capazes de reduzir desde o consumo de energia até a transmissão de doenças infecciosas ^23^. Na saúde, o investimento em pesquisa proporcionará formas de enfrentar as consequências das mudanças climáticas, com medidas que conciliem meio ambiente e saúde pública ^24^. Esses fenômenos exigem medidas preventivas, como o melhoramento dos sistemas de vigilância por meio de imagens de satélite e modelos climáticos, capazes de gerar alertas para a emergência ou a reemergência de doenças infecciosas ^25^.

Nas reuniões preparatórias para o G20, mostrou-se promissora a participação de pesquisadores de instituições renomadas na construção das agendas. Também foram elaborados Notas Temáticas (*Issues Notes)*, que são documentos de alinhamento entre os países membros do G20 com as diretrizes dos tópicos centrais. Clima, pobreza, bioeconomia, agricultura, emprego, educação, saúde e transição energética são alguns dos temas elencados.

No Notas Temáticas do Grupo de Trabalho em Saúde (*Health Working Group*), há um segmento sobre mudanças climáticas, refletindo sobre as ligações entre elas e o surgimento/propagação de doenças infecciosas nas periferias urbanas, os impactos das ondas de calor na saúde, o acesso à água potável e os impactos das alterações climáticas na saúde mental. O Brasil, assim como anteriormente fizeram Índia e Indonésia, reforça o papel da *Uma Só Saúde*, abordagem integradora na qual a saúde pública não se restringe mais apenas aos seres humanos, mas envolve todo o ambiente ^26^.

Já no G20 Força-tarefa para Mobilização Global contra a Mudança do Clima (*Task Force on a Global Mobilization against Climate Change*), são prioridades: rever medidas para a mobilização de capital privado para os países em desenvolvimento; mobilizar o financiamento público e privado para o desenvolvimento sustentável e a ação climática;- e incentivar o papel dos bancos de desenvolvimento na injeção de recursos financeiros para a mitigação e adaptação às alterações climáticas e para a implementação de Agenda 2030 ^27^.

É necessário um conjunto de ações político-econômicas capazes de combinar transição energética, ação ativa do Estado e justiça socioambiental em escala global. As mudanças climáticas requerem medidas contundentes no campo das políticas públicas e interlocução com a sociedade civil. O comércio de créditos de carbono, a suavização de índices de poluição ou a mobilização de investimentos sem contrapartidas concretas e com aferições flexíveis são insuficientes para a construção de um cenário promissor para os próximos anos.

Resta saber se a Cúpula do G20 será capaz de gerar resultados concretos. Inserida num universo cuja governança tem base no multilateralismo, os debates sobre questões como desigualdade, fome e questão ambiental se revelam fundamentais. O G20 abriga o Grupo dos Sete (G7), que reúne os países mais industrializados do mundo ocidental e atua apresentando posições duras em assuntos como financiamento, crescimento econômico e gestão de riquezas. Espera-se que, no Brasil, haja a possibilidade das medidas do G20 avançarem institucionalmente em questões prementes com a do clima. A construção de protocolos com maior grau de concretude e a adoção pactuada de medidas que ampliem a justiça socioambiental global, com articulações de financiamento fiáveis e duradouras, constituirão um grande alento para a COP-Clima, a ser realizada em Belém em 2025.

O G20 deve construir uma institucionalidade mais efetiva, com ações que incluam financiamentos condizentes com a premência que a questão apresenta. A inclusão da saúde nos documentos se mostra acertada, mas ainda carece de mecanismos claros de atuação. A simples compensação como lógica ambiental é uma espécie de indulgência ecológica para os países mais ricos, com base na compra do direito de poluir ^28^. E mesmo com iniciativas corporativas para limpar e antecipar danos e com a ação de ONGs ambientais ^29^, se vigorar a financeirização total da natureza e a racionalização dos arranjos econômicos e ambientais do ponto de vista empresarial em uma escala global, os avanços tendem a ser muito tímidos.
